# Medical School Changes

**Published:** 1849-04

**Authors:** 


### MEDICAL SCHOOL CHANGES.

The present season is likely to be a memorable one for the changes in the organization of western medical schools. At Lexington, Memphis, and Louisville there have been resignations and new appointments. Dr. Drake, who has been so long identified with medical teaching in the Valley of the Mississippi, has severed his connection with the University of Louisville, and offers his services to the public, at Cincinnati, in the practice of medicine. Dr. Bartlett and Dr. Thomas D. Mitchell have resigned their professorships in Transylvania University, and Dr. Henry M. Bullitt, of this city, has accepted the chair of Materia Medica vacated by the latter. Dr. Donne has retired from the chair of Anatomy which he accepted two years since in the Memphis Medical College.

While speaking of medical schools we ought not to fail to announce, what we are sure it will rejoice our readers to learn, that the charter for a second school in Louisville was rejected in the Senate by a decisive majority. The citizens of Louisville have cause to be gratified at the success of their present school, and are too wise, we believe, to jeopard its prosperity by attempting a second. We trust the hopeless project is now finally abandoned.

